# A Randomized Controlled Trial on the Effectiveness of Epidermal Growth Factor-Containing Ointment on the Treatment of Solar Lentigines as Adjuvant Therapy

**DOI:** 10.3390/medicina57020166

**Published:** 2021-02-13

**Authors:** Hye One Kim, Hye Ran Kim, Jin Cheol Kim, Seok Young Kang, Min Je Jung, Sung Eun Chang, Chun Wook Park, Bo Young Chung

**Affiliations:** 1 Department of Dermatology, Hallym University Kangnam Sacred Heart Hospital, Hallym University College of Medicine, Seoul 07441, Korea; hyeonekim@gmail.com (H.O.K.); cyberkhr@hanmail.net (H.R.K.); aiekfne@naver.com (J.C.K.); tjdjrdud@naver.com (S.Y.K.); luckyminja77@naver.com (M.J.J.); 2 Asan Medical Center, Department of Dermatology, Ulsan University College of Medicine, Seoul 05505, Korea; csesnumd@gmail.com

**Keywords:** pigmentary disorder, epidermal growth factor, Q-switched 532 nm Nd:YAG laser, post-inflammatory hyperpigmentation

## Abstract

*Background and Objective*: Little is known about the anti-pigmentation effects of whitening agents on solar lentigines. Epidermal growth factor (EGF) has been used as a booster for wound healing in the skin, and it has been suggested to have anti-pigmentation effects. This study aimed to evaluate the effect and safety of EGF-containing ointment for treating solar lentigines with a Q-switched (QS) 532 nm neodymium-doped yttrium aluminum garnet (Nd:YAG) laser (Bluecore company, Seoul, Republic of Korea). *Materials and Methods*: Subjects who underwent QS 532 nm Nd:YAG laser treatment of solar lentigines were randomly assigned to treatment with an EGF ointment or petrolatum. After the laser procedure, the subjects were administered the test ointment twice a day for 4 weeks. The physician’s assessment of the degree of pigment clearance and patient’s satisfaction were assessed after 4 and 8 weeks. Additionally, the melanin index (MI), erythema index (EI), transepidermal water loss (TEWL), and post-inflammatory hyperpigmentation (PIH) were evaluated. This trial was registered with ClinicalTrials.gov (NCT04704245). *Results*: The blinded physician’s assessment using 5-grade percentage improvement scale and patient’s satisfaction were significantly higher in the study group than in the control group at the 4th and 8th weeks. The MI was significantly higher in the control group than in the study group at the 4th and 8th weeks. The EI and TEWL did not differ significantly between the two groups at either time point. The incidence of PIH was higher in the control group (37.5%) than in the EGF group (7.14%) at the 8th week. *Conclusions*: The application of EGF-containing ointment on facial solar lentigines with a QS 532 nm Nd:YAG laser showed efficient and safe therapeutic effects, with less PIH. Thus, EGF-containing ointment could be suggested as the promising adjuvant treatment strategy with a QS laser for solar lentigines.

## 1. Introduction

Solar lentigines are the most common epidermal hyperpigmentary disorders in Asians. These are clinically characterized by small, well-circumscribed, round, brown-to-black-colored macules, mostly distributed in sun-exposed areas [1,2]. The chronic exposure to sun and other environmental factors such as traffic-related air pollutants (e.g., particulate matter and NO_2_ gas) may influence the development of lentigines [3,4]. Solar lentigines often appear on the face and has considerable negative impact on the quality of life [5]; therefore, many patients with lentigines and physicians consider treating this condition despite its benign features. Currently, several chemical and physical treatment methods are used for removing solar lentigines [1,6]. A topical agent containing retinoid (retinaldehyde), a phenolic agent (4-(1-phenylethyl)-resorcinol) and an antioxidant (δ-tocopheryl-β-D-glucopyranoside) showed significant efficacy in the depigmentation of solar lentigines [7]. However, a chemical treatment, such as retinoids when used alone can show unsatisfactory effects, particularly in diffused and advanced types of solar lentigines. Thus, for better therapeutic efficacy and for clinical improvement on solar lentigines, a combination of topical and procedural therapies can be performed [8].

As a physical treatment, Q-switched (QS) 532 nm neodymium-doped yttrium aluminum garnet (Nd:YAG) is a common laser treatment of solar lentigines in Asians [9]. Although a QS laser can destroy melanosomes and melanin-containing cells, this procedure also affects the surrounding vessels and produces inflammation [10]. Therefore, post-inflammatory hyperpigmentation (PIH) can appear after QS 532 nm Nd:YAG laser exposure. Especially in a darker skin type, laser therapy is often challenging because of occurrence of PIH [9,11].

Epidermal growth factor (EGF) facilitates wound healing by stimulating resurfacing of damaged epidermis and inducing granulation tissue outgrowth, angiogenesis, and wound contraction [12,13]. In actual practice, EGF is commonly used to treat chronic skin ulcers, including diabetic foot ulcers and burn injuries [14,15]. It has been reported that EGF has anti-inflammatory and antioxidant effects and directly lowers melanin production [12,13,16]. Based on this action, the possibility of the applicability of EGF on anti-pigmentation has recently been proposed.

However, there are few clinical studies regarding the therapeutic effects of EGF on pigmentary disorders. Therefore, this study aimed to evaluate the efficacy and safety of EGF-containing ointment for the treatment of solar lentigines with a QS 532 nm Nd:YAG laser.

## 2. Materials and Methods

This study was a prospective, randomized, placebo-controlled clinical trial.

### 2.1. Subjects

We enrolled 40 subjects with Fitzpatrick skin types III–IV, diagnosed by a dermatologist as having solar lentigines. The inclusion criteria for the study were as follows: (1) subjects with characteristic facial lentigines, which were typical solar lentigines that occurred after adulthood, and (2) age of 20 years or older. The exclusion criteria included the following: (1) uncontrolled systemic or chronic disease (chronic obstructive pulmonary disease, autoimmune disease, malignant tumor, etc.), (2) hypersensitivity to the ingredients of the ointment, (3) current use of skin whitening agents (hydroquinone, tretinoin, or kojic acid), (4) a history of other laser treatments or procedures for skin whitening within the past 6 months, and (5) pregnancy or lactation.

### 2.2. Study Design

Subjects were randomly assigned to groups for treatment with either an EGF-containing ointment (Easyef Saesal ointment, Daewoong Pharmaceutical Co., Ltd., Seoul, Korea) (the study group) or the vehicle alone (petrolatum; the control group). The EGF ointment included recombinant human EGF (1 μg/g). Random numbers used for assignment to groups were provided by the randomization function of SAS (version 9.3, SAS Institute, Cary, NC, USA).

The subjects received one session of laser treatment with a QS 532 nm Nd:YAG laser (Iris, Bluecore Company Co., Ltd., Busan, Korea) of their solar lentigines after enrollment. The procedure parameters were as follows: 5–10-ns pulse duration, 3.5-mm spot size, 0.9–1.1 J/cm^2^ fluence, and 2-Hz frequency. The end point of laser treatment for lentigines was immediate whitening. The subjects then applied the EGF ointment or vehicle twice daily (morning and evening) to the lesion for 4 weeks after laser treatment. This study was approved by the institutional review board of Hallym University Kangnam Sacred Heart Hospital (IRB no. 2017-06-009). This study was registered with ClinicalTrials.gov, reference NCT04704245.

### 2.3. Outcome Measurement

#### 2.3.1. Physician’s Evaluation

At 4 and 8 weeks after EGF ointment or petrolatum application, the pigment clearance was assessed using a 5-grade percentage improvement scale (grade 1, <0% (worse); grade 2, 0–25% improvement; grade 3, 26–50% improvement; grade 4, 51–75% improvement; grade 5, 76–100% improvement) [17].

Two blinded independent dermatologists reviewed the photographs of before and after treatment and evaluated the grade of pigment clearance using a 5-grade percentage improvement scale.

#### 2.3.2. Objective Evaluation of Melanin Index, Erythema Index, and Transepidermal Water Loss

A spectrophotometer and VapoMeter^®^ (Delfin Technologies Ltd., Kuopio, Finland) were used to assess the lesion in a room at constant temperature (20–24 °C) and humidity (28–38%) at the 2nd, 4th, and 8th weeks of the clinical trial. Skin measurements were performed using an instrument to determine erythema (erythema index, EI), pigmentation (melanin index, MI), and transepidermal water loss (TEWL). We measured the TEWL for evaluating skin barrier function or integrity [18,19].

#### 2.3.3. The Patient’s Subjective Evaluation

The patient’s subjective satisfaction was assessed on weeks 4 and 8 according to the following: 1, worse; 2, no change; 3, mild improvement; 4, moderate improvement; or 5, significant improvement.

#### 2.3.4. PIH Measurement

The presence of PIH at the laser treatment site was evaluated at weeks 4 and 8. PIH was defined as a case in which the MI of the lesion at the end of the study was higher than that measured before laser treatment [17].

### 2.4. Statistical Analysis

All data are expressed as mean ± standard deviation. The demographic and clinical characteristics, along with differences in the MI, EI, and TEWL of the two groups, were evaluated using Student’s *t*-test and the paired *t*-test. The difference in PIH incidence between the two groups was evaluated using Fisher’s exact test. A *p*-value < 0.05 was considered statistically significant. All statistical analyses were performed using IBM SPSS Statistics 24.0 (IBM Co., Armonk, NY, USA).

## 3. Results

### 3.1. Baseline Characteristics of the Subjects

**Figure 1:**
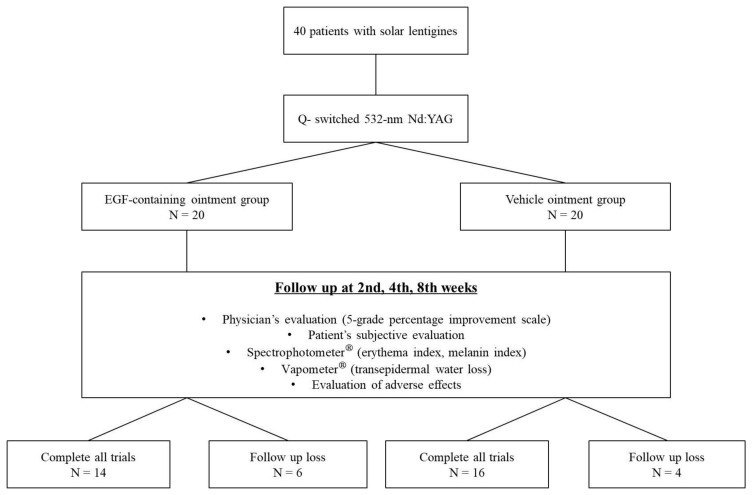
Flow chart of the study participant inclusion. A total of 40 subjects (20 in the study group and 20 in the control group) were enrolled in this study, and 30 (14 in the study group and 16 in the control group) completed all clinical evaluations (excluding those who were lost to follow-up).

A total of 40 subjects (20 in the study group and 20 in the control group) were enrolled in this study, and 30 (14 in the study group and 16 in the control group) completed all clinical trials, after 10 subjects were lost to follow-up (Figure 1).

The mean age of the study group was 56.71 (±2.62) years, with a female-to-male ratio of 11:3, and that of the control group was 58.63 (±2.53) years, with a female-to-male ratio of 11:5. The Fitzpatrick skin types were III (*n* = 2) and IV (*n* = 12) in the study group and III (*n* = 3) and IV (*n* = 13) in the control group. There were no statistically significant differences in the mean age or mean MI, EI, and TEWL values between the two groups in baseline (Table 1).

### 3.2. Physician’s Assessment

**Figure 2:**
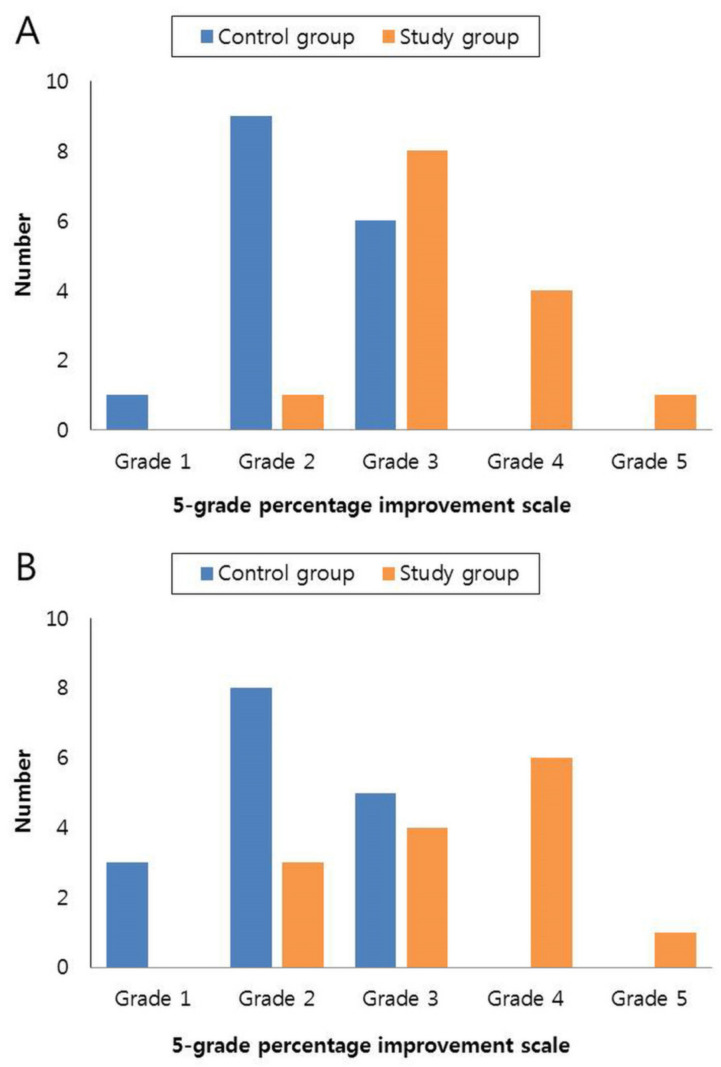
(**A**) Assessment of the 5-grade scale for improvement by a physician at 4 weeks. (**B**) Assessment of the 5-grade scale for improvement by a physician at 8 weeks.

**Figure 3:**
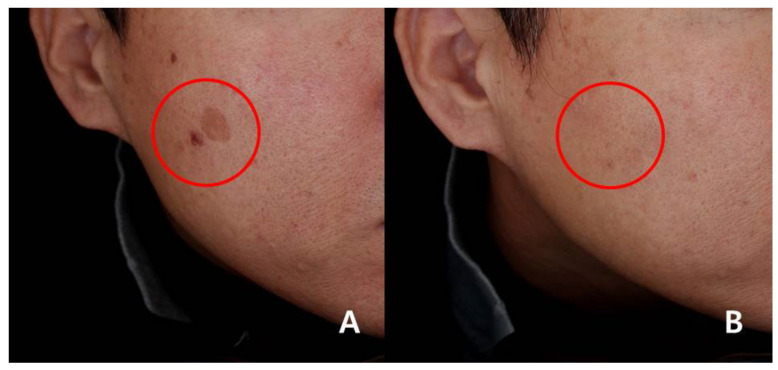
Clinical photographs of the patients in the study group (grade 5 improvement). (**A**) Before treatment in the study group. (**B**) At 8 weeks in the study group. Red circles; treated areas.

**Figure 4:**
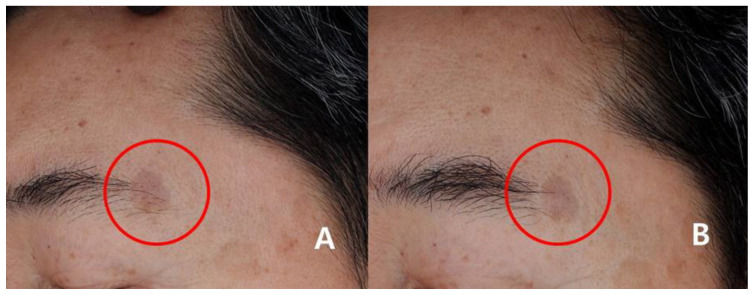
Clinical photographs of the patients in the control group (grade 2 improvement). (**A**) Before treatment in the control group. (**B**) At 8 weeks in the control group. Red circles; treated areas.

In the study group, 92.85% (13/14) at week 4 and 78.57% (11/14) at week 8 were assessed as grade 3 or higher. In contrast, in the control group, 37.5% (6/16) at week 4 and 31.25% (5/16) at week 8 were assessed as grade 3 or higher (Figure 2A,B). The clinical examples of both groups are shown in Figure 3 and Figure 4.

### 3.3. MI, EI, and TEWL

**Figure 5:**
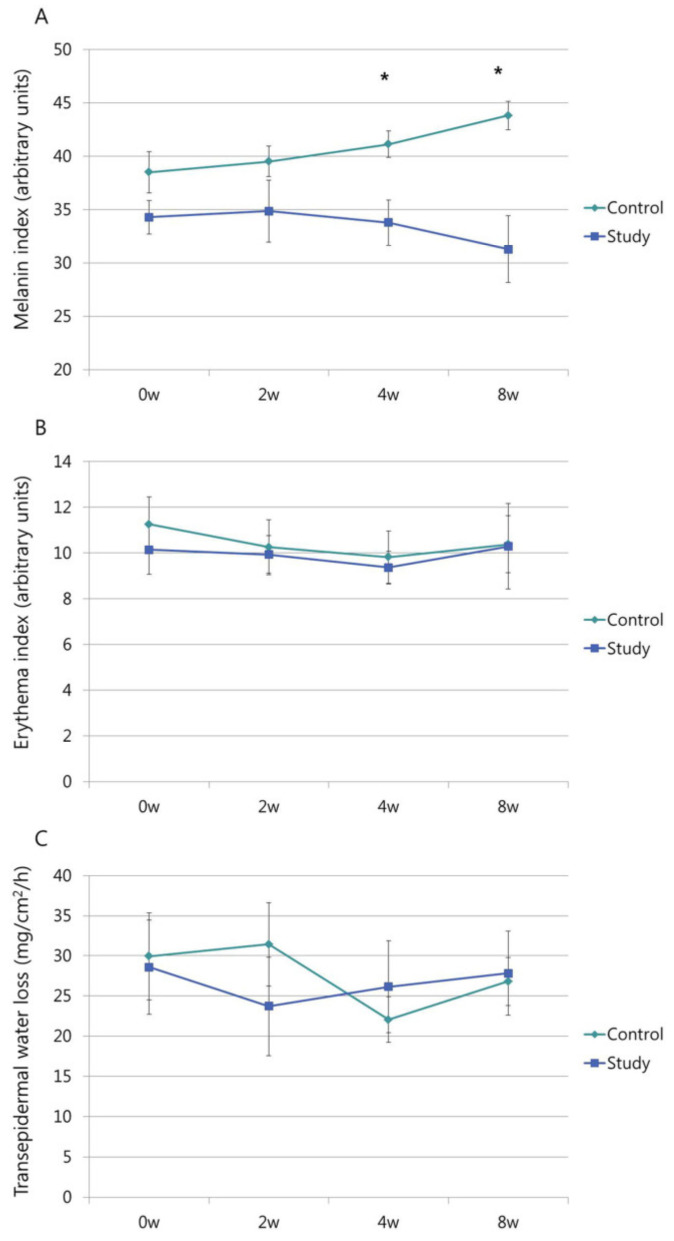
(**A**) Melanin index (MI) at baseline and follow-up observation. At weeks 4 and 8, the control group showed significantly higher MI than the study group. * *p* < 0.05 compared with the control group. (**B**) Erythema index (EI) at baseline and follow-up observation. The EI measured at week 8 showed no significant difference between the groups (*p* = 0.968). Both the study group (*p* = 0.941) and control group (*p* = 0.405) showed no significant difference between the baseline and 8-week EI values. (**C**) Transepidermal water loss (TEWL) at baseline and follow-up observation. For TEWL, there was no significant difference between the study and control groups at the last visit (*p* = 0.862). Both the study group (*p* = 0.911) and control group (*p* = 0.577) showed no significant difference between the baseline and 8-week TEWL values.

For the MI at weeks 4 (33.79 ± 2.12 vs. 41.13 ± 1.23, *p* = 0.005) and 8 (31.29 ± 3.11 vs. 43.81 ± 5.29, *p* = 0.001), the control group showed significantly higher MI than the study group (Figure 5A).

There was no significant change in the baseline MI and 8-week MI in the study group, but there was a significant increase in the MI over this time period in the control group (*p* = 0.023). There was a significant increase in the 8-week MI compared with the baseline MI in the control group (38.50 ± 7.78 vs. 43.81 ± 5.29, *p* = 0.01). The EI measured at the last visit showed no significant difference between the study group (10.38 ± 1.25) and control group (10.29 ± 1.88) (*p* = 0.968) (Figure 5B). Neither the study group (*p* = 0.941) nor the control group (*p* = 0.405) showed any significant difference between the baseline EI and 8-week EI. For TEWL, there was also no significant difference between the study group (26.81 ± 2.99) and control group (27.84 ± 5.26) at the last visit (*p* = 0.862). Both the study group (*p* = 0.911) and control group (*p* = 0.577) showed no significant difference between baseline TEWL and 8-week TEWL (Figure 5C).

### 3.4. Patient’s Satisfaction

Table 2 shows the distribution of the subjective satisfaction of the subjects evaluated at weeks 4 and 8. At week 4, 92.86% (13/14) of subjects in the study group reported experiencing their condition as “improved” or better. At 4 weeks, in the control group, 56.25% (9/16) reported no change in the condition, and 6.25% (1/16) reported worsening. At week 8, 78.57% (11/14) of the subjects in the study group reported “improved”, whereas 31.25% (5/16) in the control group reported “improved”.

### 3.5. Incidence of PIH and Other Adverse Effects

The incidences of PIH at the 8th week were 37.5% and 7.14% in the control (6/16) and study (1/14) groups, respectively, which were significantly different (*p* = 0.014).

None of the subjects complained of adverse effects after the application of the EGF ointment or petrolatum.

## 4. Discussion

Solar lentigines, which are macular hyperpigmented skin lesions, are a common pigmentary disorder in aged patients, caused by chronic exposure to ultraviolet irradiation [20]. The pathogenesis of the pigmentation in solar lentigines is regulated by complex pathways in a hormonal, autocrine, paracrine, or intracrine manner, leading to the upregulation of tyrosinase, the key enzyme of melanogenesis [21]. Recently, various melanogenic growth factors and cytokines regarding hyperpigmentation in solar lentigines, including stem cell factor, granulocyte–macrophage colony-stimulating factor, basic fibroblast growth factor, keratinocyte growth factor, prostaglandin E2 (PGE2), interleukin-1α, and tumor necrosis factor-α, have been identified [22,23,24,25]. However, the exact mechanism of melanocyte activation in solar lentigines is still unclear.

Although solar lentigines are the most common distressing hyperpigmentary disorders in Asians, there have been few studies regarding the effects of topical anti-pigmentation agents for these disorders [26,27,28,29]. EGF plays the critical role in regulating the dedifferentiation of keratinocytes to an epithelial linage and reestablishing the epithelial barrier [30]. Thus, EGF is important for proper and efficient wound healing. Several studies have established EGF as an effective wound healing booster [31,32]. Recent research highlight the action of growth factors on the melanogenesis [24,25]. Additionally, the evidence that EGF is involved in the regulation of melanogenesis have been suggested [12,13]. The mechanism of the inhibitory effect of EGF on pigmentation remains unclear. An in vitro study showed the anti-melanogenic effect of EGF via suppression of tyrosinase activity [13]. Furthermore, EGF may have inhibitory effects on melanogenesis by reduction of inflammation and promotion of effects on the regeneration of the epidermal barrier in damaged tissues [33,34]. The antioxidant effect of EGF could also induce anti-melanogenesis [16,35]. However, there are few clinical studies evaluating the anti-pigmentation effect of EGF on pigmentary disorders [12]. This prospective, randomized, placebo-controlled clinical study demonstrated that the application of EGF ointment could enhance the removal of facial lentigines with QS 532 nm Nd:YAG treatment more effectively, showing greater improvement in the clinician’s evaluation and the subjective satisfaction of the subjects.

Lentigines are often treated with laser to achieve cosmetic objectives, and QS 532 nm Nd:YAG is the preferred laser for use in this process [1,36]. The 532 nm wavelength emitted by Nd:YAG laser is well absorbed by melanin. Furthermore, a main therapeutic target of solar lentigines, melanosomes, can be destroyed selectively by QS lasers because the pulse widths are shorter than the thermal relaxation time of melanosomes following the principle of selective photothermolysis [10]. However, side effects occur after laser treatment, such as PIH, particularly in darker skin types, which make laser treatment challenging [37]. Post-laser PIH has been reported at the rates of 10–47% [9,38,39,40]. There have been several reports of attempts to prevent laser treatment-associated PIH [41,42,43]. For example, 10% glycolic acid cream, 4% hydroquinone cream, and 0.025% tretinoin cream have been used with CO_2_ lasers, but such treatments did not prevent PIH [41]. Oral tranexamic acid also did not prevent QS 532 nm Nd:YAG laser-induced PIH [39]. There has been only a case report regarding the α-adrenergic receptor agonist brimonidine tartrate gel plus topical steroid for the prevention of laser therapy-related PIH [40]. The present clinical study found the preventive effect of EGF in post-laser PIH consistent with the previous single study [12]. In addition, in this study, among subjects with PIH, the mean increase in MI was also lower in the study group than in the control group from the 4th week. The previous study using the PIH in vitro model reported that EGF reduced laser-induced melanin production, suggesting that EGF regulates the pro-inflammatory cytokines, including PGE2, generated by laser induced-damaged keratinocytes [13].

The limitations of this study were that the study was conducted in a single institution and it may be difficult to generalize because of the relatively small number of subjects. In this regard, further studies with more subjects are needed.

## 5. Conclusions

In conclusion, we found that the application of EGF-containing ointment treatment for removing facial solar lentigines with a QS 532 nm Nd:YAG laser showed the superior therapeutic effects in the study group compared with the vehicle group. EGF-containing ointment application could be a safe and effective adjuvant strategy for the treatment of solar lentigines with a QS 532 nm Nd:YAG laser, showing the synergistic therapeutic effect and prevention of PIH.

## Figures and Tables

**Table 1:** Baseline characteristics of subjects.

	Control Group	Study Group	*p*-Value
Number	16	14	
Sex (M/F)	5/11	3/11	
Age (years)	58.63 ± 2.53	56.71 ± 2.62	0.605
MI	38.5 ± 1.95	34.29 ± 1.57	0.109
EI	11.25 ± 1.20	10.14 ± 4.06	0.504
TEWL	29.94 ± 5.44	28.59 ± 5.90	0.867

Abbreviations: EI, erythema index; MI, melanin index; TEWL, transepidermal water loss.

**Table 2:** Patient’s satisfaction at 4 and 8 weeks.

	4 Weeks		8 Weeks	
	Control Group	Study Group	Control Group	Study Group
Worse	1 (6.25%)	0	3 (18.75%)	0
No change	9 (56.25%)	1 (7.14%)	8 (50%)	3 (21.43%)
Improved	6 (37.5%)	8 (57.14%)	5 (31.25%)	4 (28.57%)
Much improved	0	4 (28.57%)	0	6 (42.86%)
Very much improved	0	1 (7.14%)	0	1 (7.14%)

## Data Availability

The data used and analyzed during the present study are available from the corresponding author upon reasonable request. The data are not publicly available due to possible personal information breaches though they were de-linked.
